# Integrated transcriptomic and metabolomic analysis reveals the effects of polyploidization on the lignin content and metabolic pathway in *Eucalyptus*

**DOI:** 10.1186/s13068-023-02366-4

**Published:** 2023-07-21

**Authors:** Tingting Xu, Zhao Liu, Dingju Zhan, Zhenwu Pang, Shuwen Zhang, Chenhe Li, Xiangyang Kang, Jun Yang

**Affiliations:** 1 State Key Laboratory of Tree Genetics and Breeding, National Engineering Research Center of Tree Breeding and Ecological Restoration, Key Laboratory of Genetics and Breeding in Forest Trees and Ornamental Plants of Ministry of Education, College of Biological Sciences and Technology, Beijing Forestry University, Beijing, 100083 China; 2 Guangxi Bagui R&D Institute for Forest Tree and Flower Breeding, Nanning, 530025 China

**Keywords:** *Eucalyptus*, Lignin, Polyploidization, Transcription factor, Triploid, Co-expression network

## Abstract

**Background:**

Lignin is a major restriction factor for the industrial production of biomass resources, such as pulp and bioenergy. *Eucalyptus* is one of the most important sources of pulp and bioenergy. After polyploidization, the lignin content of forest trees is generally reduced, which is considered a beneficial genetic improvement. However, the differences in the lignin content between triploid and diploid *Eucalyptus* and the underlying regulatory mechanism are still unclear.

**Results:**

We conducted a comprehensive analysis at the phenotypic, transcriptional and metabolite levels between *Eucalyptus urophylla* triploids and diploids to reveal the effects of polyploidization on the lignin content and lignin metabolic pathway. The results showed that the lignin content of *Eucalyptus urophylla* triploid stems was significantly lower than that of diploids. Lignin-related metabolites were differentially accumulated between triploids and diploids, among which coniferaldehyde, *p*-coumaryl alcohol, sinapaldehyde and coniferyl alcohol had significant positive correlations with lignin content, indicating that they might be primarily contributing metabolites. Most lignin biosynthetic genes were significantly downregulated, among which 11 genes were significantly positively correlated with the lignin content and above metabolites. Furthermore, we constructed a co-expression network between lignin biosynthetic genes and transcription factors based on weighted gene co-expression network analysis. The network identified some putative orthologues of secondary cell wall (SCW)-related transcription factors, among which *MYB52*, *MYB42*, *NAC076*, and *LBD15* were significantly downregulated in *Eucalyptus urophylla* triploids. In addition, potential important transcription factors, including *HSL1*, *BEE3*, *HHO3*, and *NAC046*, also had high degrees of connectivity and high edge weights with lignin biosynthetic genes, indicating that they might also be involved in the variation of lignin accumulation between triploid and diploid *Eucalyptus urophylla*.

**Conclusions:**

The results demonstrated that some lignin-related metabolites, lignin biosynthetic genes and transcription factors in *Eucalyptus urophylla* triploids may be relatively sensitive in response to the polyploidization effect, significantly changing their expression levels, which ultimately correlated with the varied lignin content. The analysis of the underlying formation mechanism could provide beneficial information for the development and utilization of polyploid biomass resources, which will be also valuable for genetic improvement in other bioenergy plants.

**Supplementary Information:**

The online version contains supplementary material available at 10.1186/s13068-023-02366-4.

## Background

The sustainability of woody biomass makes it an important lignocellulosic resource*.* However, the main limitation of using such biomass for industrial production is the recalcitrance of wood to degradation, which mainly depends on the content and composition of lignin deposited in secondary cell walls (SCWs) [1, 2]. The total lignin content and composition vary in distinct species and different tissues or developmental stages within the same plant [3]. These variations can be regulated by different molecular mechanisms in plants. Polyploid induction is commonly employed to generate novel traits that are useful in forest tree breeding programs [3–5]. Several studies have shown that cell wall components can be altered in polyploids, among which the lignin content tends to decrease in multiple polyploid plants compared with that of diploids, such as poplar, shrub willow, *Arabidopsis*, rice, potato, etc. [6–11]. The variation of low lignin content in polyploids may better meet the demands for the production and utilization of lignocellulosic biomass.

As one of the top three timber species in the world, eucalypt are well-known for their rapid growth, superior wood properties and outstanding adaptability and are widely planted worldwide [12]. Its wood is extensively used for pulp, bioenergy production, etc., resulting in great economic value [13, 14]. To improve the growth rate and biomass accumulation, triploid *Eucalyptus urophylla* has been successfully induced and showed a significant growth advantage over diploid *Eucalyptus urophylla* [15, 16]. However, the effects of polyploidization on the lignin content and metabolic pathway have not yet been reported.

Studies have shown that many genes are differentially expressed after polyploidization [17]. These differentially expressed genes (DEGs) are involved in various physiological processes in plants, leading to the formation of polyploid phenotypic variations [18]. As an important secondary metabolite in plants, the accumulation of lignin is a complex process involving multiple pathways, which require the participation of various enzymes and their encoding genes [19]. Specifically, three upstream enzymes, PAL (phenylalanine ammonia-lyase), C4H (cinnamate 4-hydroxylase) and 4CL (4-coumarate: CoA ligase), are involved in the synthesis of various phenylpropanoid compounds in the phenylpropanoid metabolic pathway (Fig. 3B). After a series of catalytic reactions, three monomers, *p*-coumaryl alcohol, coniferyl alcohol and sinapyl alcohol, are generated through the monolignol specific synthesis pathway, which includes enzymes of C3′H (*p*-coumaroyl shikimate-3-hydroxylase), HCT (*p*-hydroxycinnamoyl-CoA: quinate/shikimate), CSE (caffeoyl shikimate esterase), COMT (caffeic acid *O*-methyltransferase), CCoAOMT (caffeoyl-CoA-3-*O*- methyltransferase), F5H (ferulate 5-hydroxylase), CCR (cinnamoyl-CoA reductase) and CAD (cinnamyl alcohol dehydrogenase) (Fig. 3B). Finally, three monomers are oxidatively polymerized to corresponding lignin units by LAC (laccases) and POD (peroxidases) (Fig. 3B). In addition, lignin synthesis is also regulated by a large complex transcriptional network, and many transcription factors (TFs) have been identified that regulate the process [20, 21]. These important enzyme-encoding genes and key regulatory factors may be affected by polyploidization, altering their expressions and regulation patterns; however, the above process has not been elucidated in *Eucalyptus urophylla*.

Multi-omics analysis is a more comprehensive method for revealing the mechanism underlying trait formation. Transcriptome sequencing has been widely used to identify genes related to target traits, and metabolic profiling can be used to study metabolites in an organism at a particular point in time [22–24]. In this study, integrated transcriptomic and metabolomic analyses were performed to identify the differences in lignin accumulation between *Eucalyptus urophylla* triploids and diploids. Key genes and metabolites related to lignin were identified, and their expression patterns were analyzed. Furthermore, a co-expression network was constructed to identify higher level regulatory factors. These results provide a better understanding of the effects of polyploidization on the lignin content and underlying regulatory mechanisms between *Eucalyptus urophylla* triploids and diploids. This study also provides an important theoretical basis for the development and utilization of polyploid biomass resources.

## Results

### Analysis of the lignin content in triploid and diploid stems of *Eucalyptus urophylla*

**Fig. 1:**
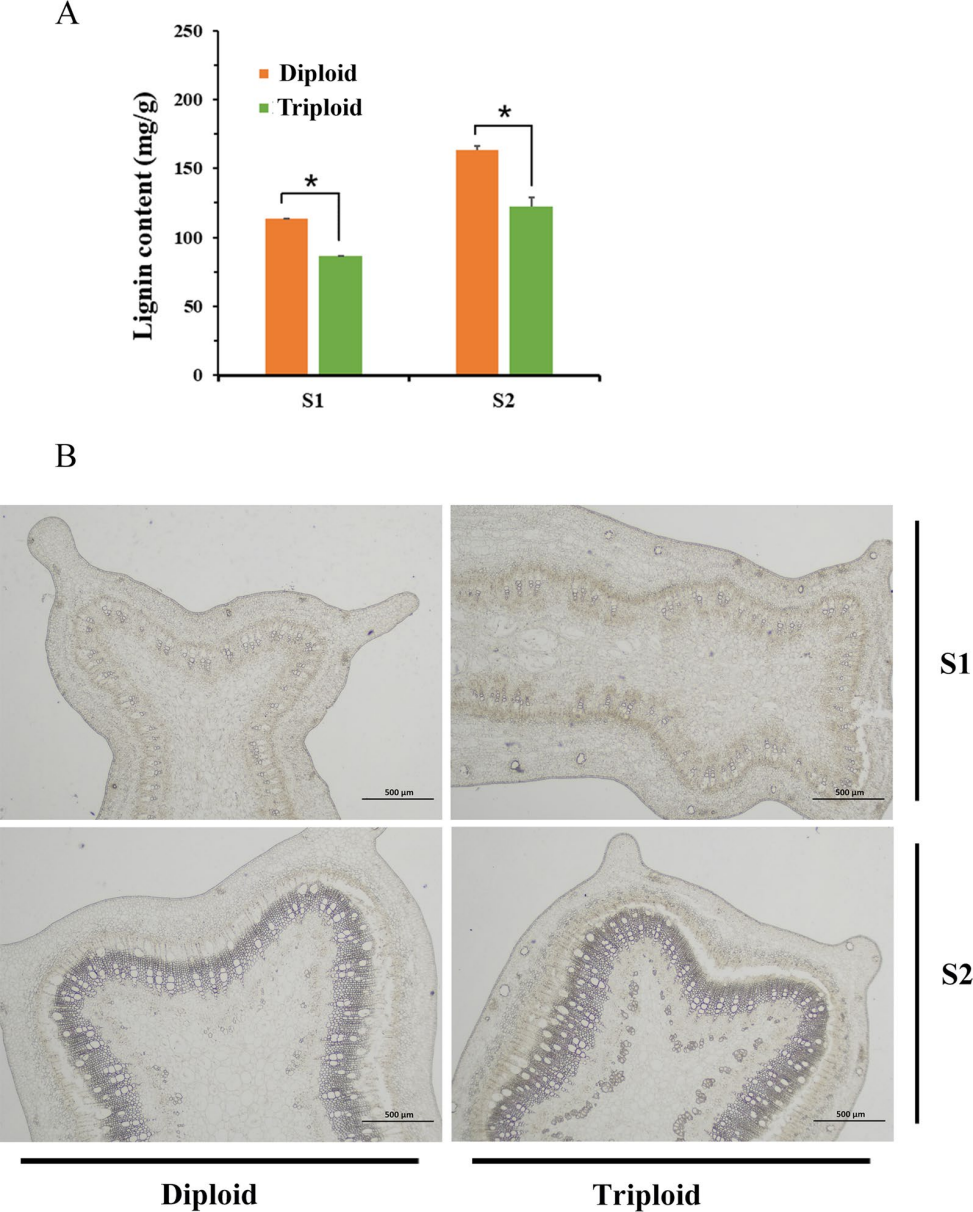
Analysis of *Eucalyptus urophylla* triploid and diploid stems. **A** Lignin content of stems in two different developmental stages. **B** Cross sections of stems in two different developmental stages. An asterisk indicates that there is a significant difference between triploids and diploids, which was determined by Student’s *t*-test (*p* < 0.05). Error bars indicate the standard deviation among the three replicates

Lignin synthesis mainly occurs during the secondary wall thickening of xylem cells in plants. In this study, we measured and analyzed the lignin content of stems in two different developmental stages between *Eucalyptus urophylla* triploids and diploids. The results showed that the lignin content in triploid stems was significantly lower than that of diploids in both developmental stages (Fig. 1A). In this study, stems at both developmental stages are represented by S1 and S2, respectively. S1 was the relatively tender top part of the stem. Histochemical staining of the cross section showed that a small number of secondary cell walls were deposited and colored (Fig. 1B). The cross section of S2 showed that the xylem was connected into a ring (Fig. 1B). Lignification was higher in S2. These results suggested that the lignin accumulation of *Eucalyptus urophylla* triploids was significantly reduced compared with that of diploids in different growth stages of secondary wall synthesis.

### General transcriptome profiling of *Eucalyptus urophylla* triploids and diploids

**Fig. 2:**
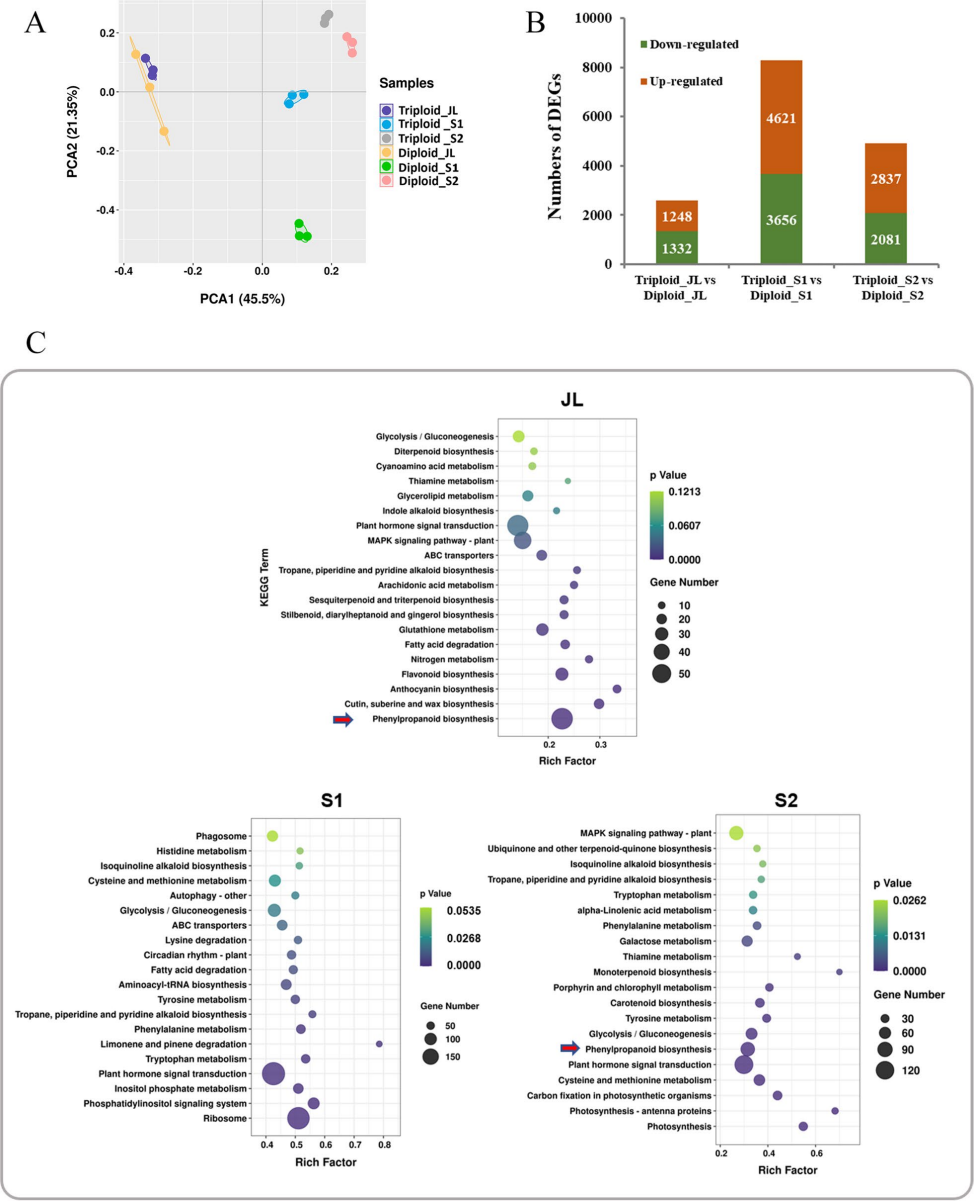
General transcriptome analysis of *Eucalyptus urophylla* triploids and diploids. **A** Principal component analysis of transcriptome data. **B** Numbers of differentially expressed genes (DEGs) between *Eucalyptus urophylla* triploids and diploids. **C** Pathway enrichment analysis of DEGs between *Eucalyptus* triploids and diploids. The *Y*-axis indicates the KEGG pathway; the *X*-axis indicates the rich factor. The dot size indicates the number of DEGs of the pathway, and the dot color indicates the *p*-value

To analyze gene expression changes between *Eucalyptus urophylla* triploids and diploids, we separated stems from two different developmental stages (S1 and S2) and juvenile leaves (JL) from 4-month-old plants to perform transcriptome sequencing. A total of 117.23 GB of clean data were obtained from 18 samples, including three replicates, thus accounting for more than 88.42% of the raw reads. The percentage of Q30 in each sample was above 97.1% (Additional File 5: Table S2). Thus, the quality of these data was suitable to conduct further bioinformatic analysis. The principal component analysis based on the expression of all transcripts showed that samples with different ploidies or from different tissues had different gene expression patterns. A high degree of similarity was observed among the replicates of each genotype (Fig. 2A).

An analysis of DEGs showed that the numbers of DEGs were the highest in S1 and the lowest in JL between *Eucalyptus urophylla* triploids and diploids (Fig. 2B). To explore the biological pathways DEGs involved, we conducted the Kyoto Encyclopedia of Genes and Genomes (KEGG) enrichment analysis. In JL, the DEGs were most significantly enriched in “phenylpropanoid biosynthesis” (kegg00940), “cutin, biosynthesis of keratin, suberine, and wax” (kegg00073) and “flavonoid biosynthesis” (kegg00941) between *Eucalyptus urophylla* triploids and diploids (Fig. 2C); in S1, the DEGs were most significantly enriched in “ribosome” (kegg03010), “phosphatidylinositol signaling system” (kegg04070) and “plant hormone signal transduction” (kegg04075) between *Eucalyptus urophylla* triploids and diploids (Fig. 2C); and in S2, the DEGs were most significantly enriched in “photosynthesis” (kegg00195), “carbon fixation in photosynthetic organisms” (kegg00710) and “metabolism of cysteine and methionine” (kegg00270) between *Eucalyptus urophylla* triploids and diploids (Fig. 2C). In addition, in S2, the “phenylpropanoid biosynthesis” (kegg00940) pathway related to lignin biosynthesis was also significantly enriched (Fig. 2C).

### Identification and analysis of differentially expressed lignin-related genes between *Eucalyptus urophylla* triploids and diploids

**Fig. 3:**
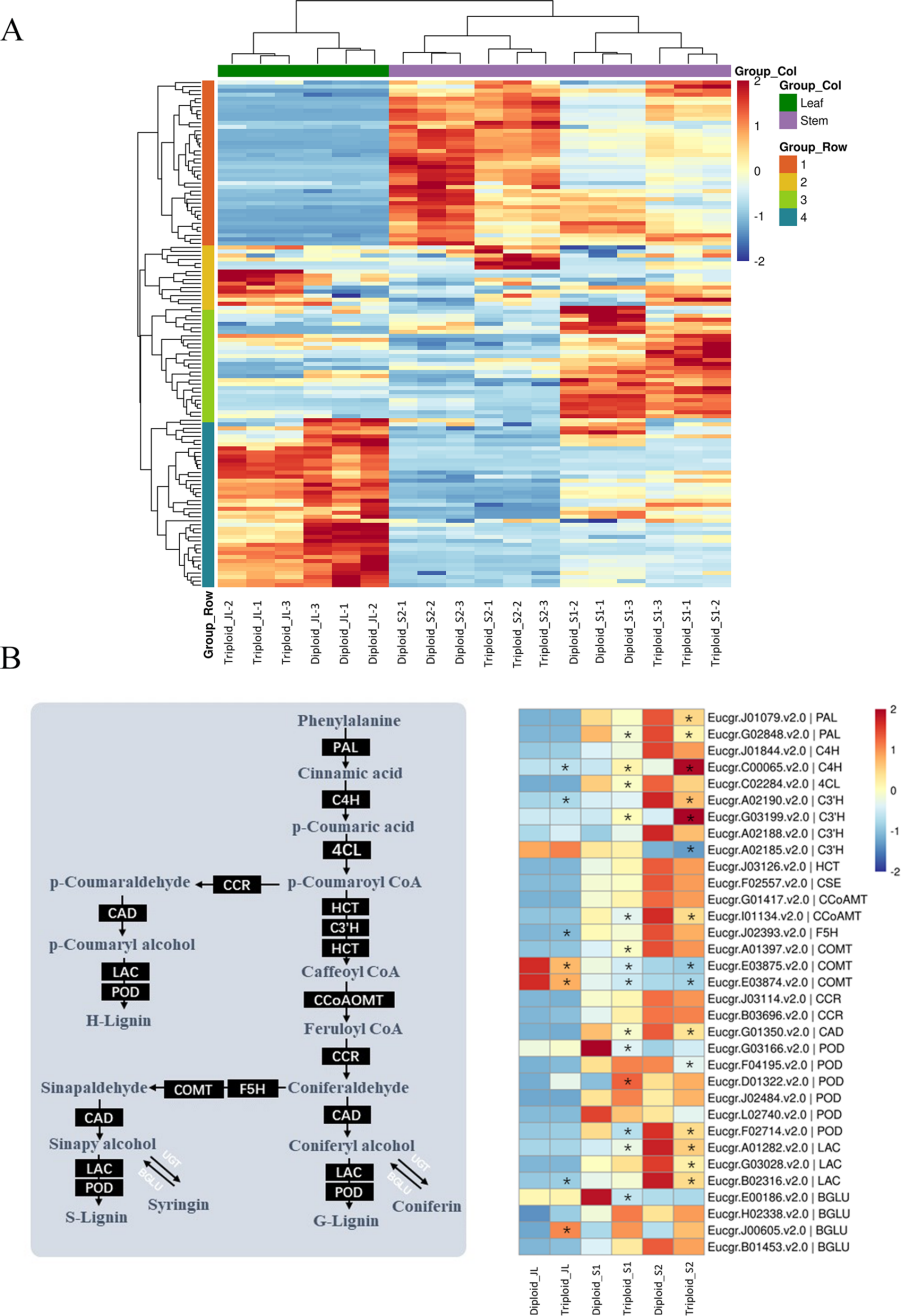
Expression analysis of lignin-related genes in *Eucalyptus urophylla* triploids and diploids. **A** Cluster heatmap analysis of lignin-related genes. **B** Differentially expressed analysis of lignin-related genes identified between *Eucalyptus urophylla* triploids and diploids. The values in the heatmap represent the z-score of the FPKM in different samples

Genes related to lignin biosynthesis mostly exist in the form of multiple gene families [25]. From the transcriptome data of this study, a total of 125 genes annotated to lignin metabolic pathway were screened. A cluster heatmap analysis was performed to identify the expression patterns of genes in different tissues and different ploidies. The results showed that the expression patterns of lignin-related genes in stems were different from those in leaves (Fig. 3A). The lignin-related genes were mainly clustered into four groups based on their expression level in different samples (Fig. 3A). The genes in Group 1 had a specific high expression in stems, which may be the primary genes involved in the lignin biosynthesis of stems. In addition, several genes in other groups with nonspecific high expression in stems were also selected for further analysis. Finally, 33 genes from 14 gene families were identified for further analysis (Fig. 3B).

The expression patterns of 33 selected genes were further analyzed, and a total of 22 genes were differentially expressed in at least one tissue between *Eucalyptus urophylla* triploids and diploids. In the general phenylpropanoid biosynthesis pathway, two *PAL* (*Eucgr.J01079.v2.0*; *Eucgr.G02848.v2.0*) were significantly downregulated in S1 and S2, *C4H* (*Eucgr.C00065.v2.0*) was significantly upregulated in the three tissues, and *4CL* (*Eucgr.C02284.v2.0*) was significantly downregulated in S1 of triploids compared with diploids. In the monolignol synthesis pathway, three *C3’Hs* were differentially expressed and two (*Eucgr.A02190.v2.0*; *Eucgr.A02185.v2.0*) were significantly downregulated in S2. *CCoAOMT* (*Eucgr.I01134.v2.0*) was significantly downregulated in S1 and S2 of triploids compared with diploids. Three *COMTs* were differentially expressed, with two (*Eucgr.E03875.v2.0*; *Eucgr.E03874.v2.0*) significantly downregulated in the three tissues and one (*Eucgr.A01397.v2.0*) differentially upregulated in S1. *CAD* (*Eucgr.G01350.v2.0*) was also significantly downregulated in S1 and S2 of triploids compared with diploids. In addition, three *LACs* (*Eucgr.A01282.v2.0*; *Eucgr.G03028.v2.0*; *Eucgr.B02316.v2.0*) related to monolignol polymerization were significantly downregulated in S2. Four *PODs* were also differentially expressed, with three (*Eucgr.G03166.v2.0*; *Eucgr.F04195.v2.0*; *Eucgr.F02714.v2.0*) differentially downregulated in S1 or S2 and one (*Eucgr.C01322.v2.0*) differentially upregulated in S1. *BGLU* (*Eucgr.E00186.v2.0*) is related to monolignol modification and was differentially downregulated in S1, and *BGLU* (*Eucgr.J00605.v2.0*) was differentially upregulated in JL.

In general, DEGs are involved in each pathway of lignin biosynthesis, and most DEGs are significantly downregulated in triploids compared with diploids, which may eventually lead to less lignin accumulation in triploid stems. To verify the expression pattern of these genes, we randomly selected several genes for determining the relative expression by qRT-PCR. The results showed a relatively consistent tendency between the transcriptomic data and qRT-PCR data (Additional file 1: Fig. S1).

### General metabolic profiling of *Eucalyptus urophylla* triploids and diploids

**Fig. 4:**
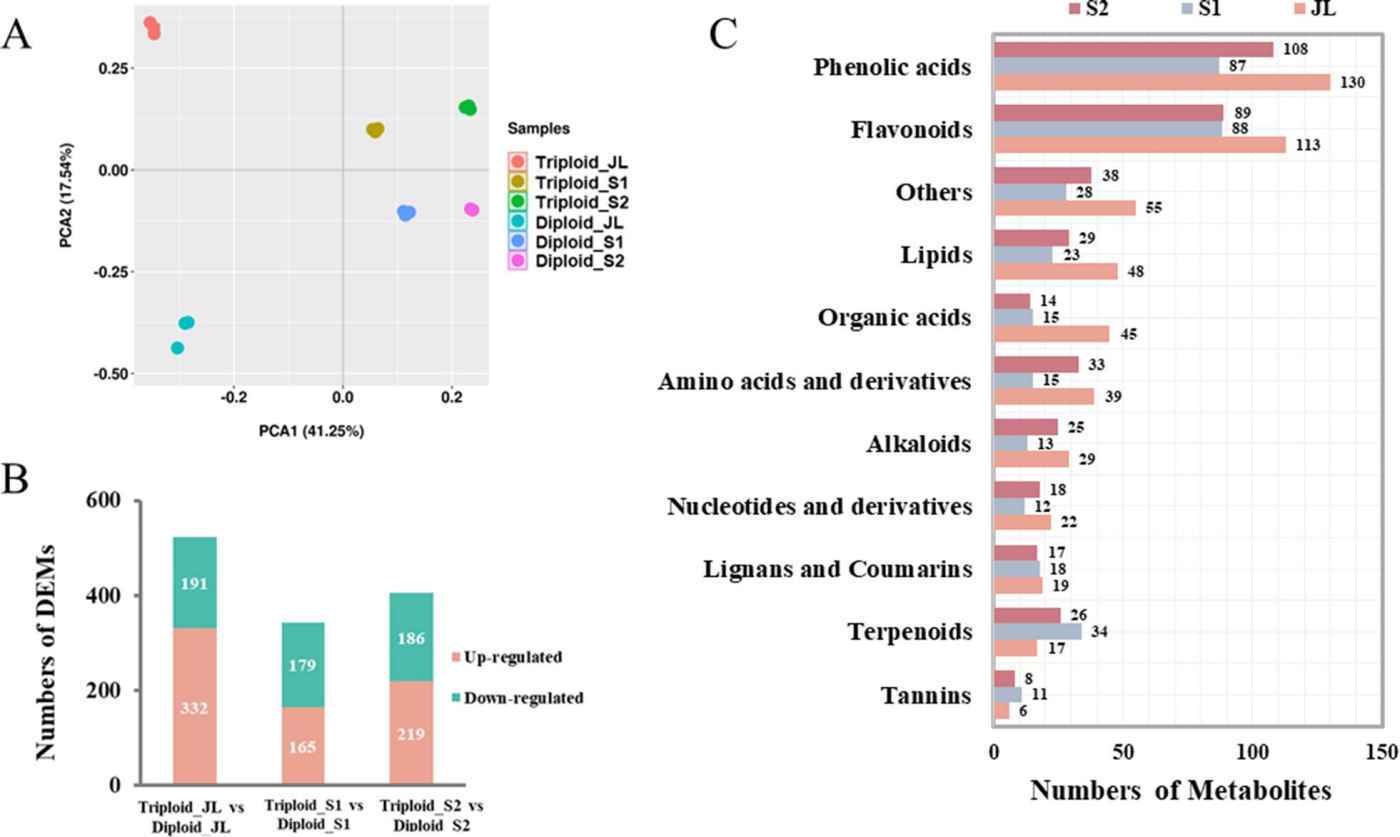
General metabolic profiling of *Eucalyptus urophylla* triploids and diploids. **A** Principal component analysis of all metabolites. **B** Numbers of differentially expressed metabolites (DEMs) between *Eucalyptus urophylla* triploids and diploids. **C** Primary classification of DEMs between *Eucalyptus urophylla* triploids and diploids

Metabolites, as a bridge between gene expression and phenotypes, are helpful for elucidating the mechanisms underlying phenotype variation. In this study, we performed metabolic profiling using the same samples for transcriptome sequencing. Principal component analysis according to the relative expression of all metabolites showed that there were small differences among three replicates (Fig. 4A). The different ploidies and different tissues relatively grouped, indicating that metabolite accumulation varied in different ploidies and tissues (Fig. 4A).

**Fig. 5:**
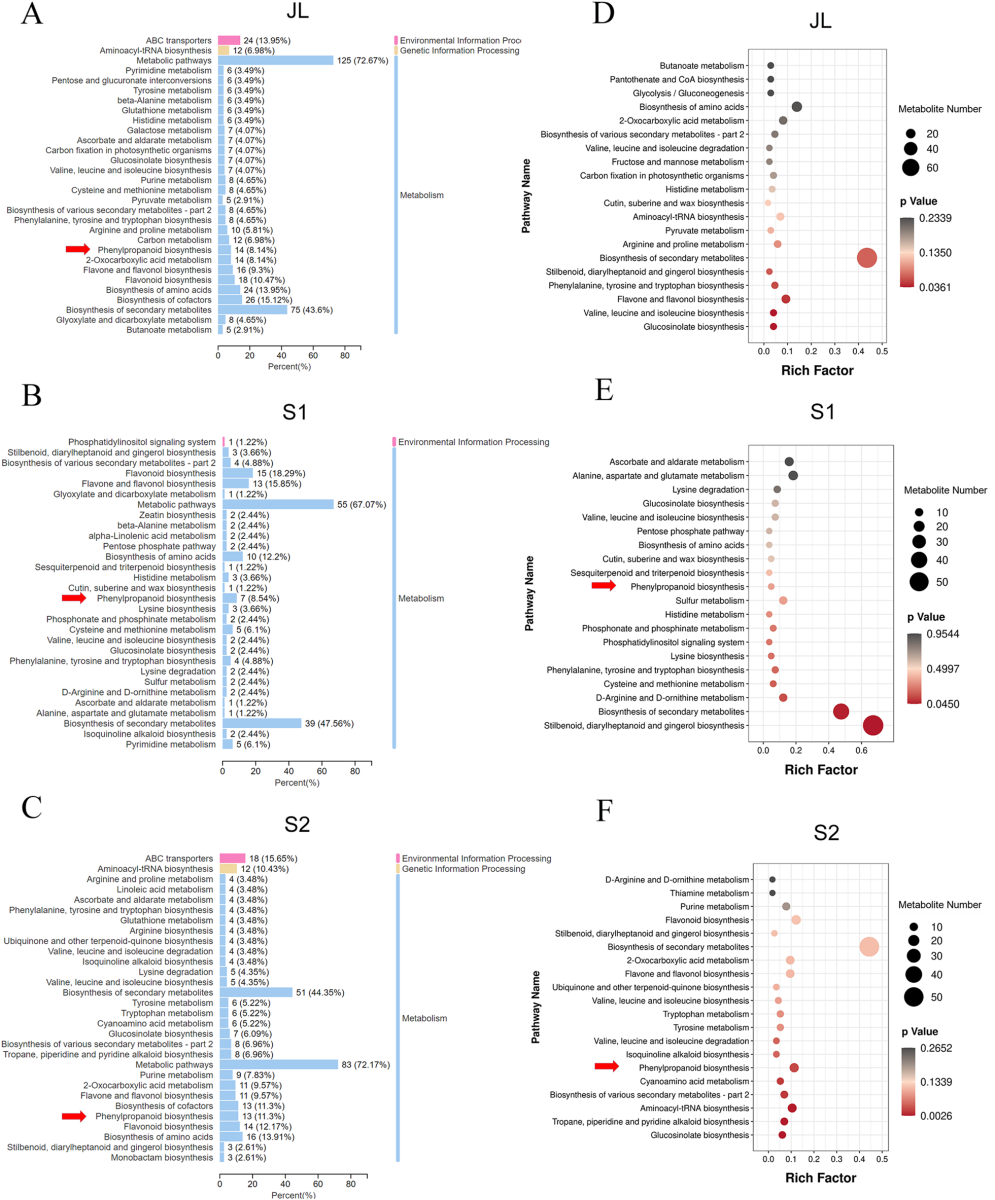
Pathway analysis of DEMs involved between *Eucalyptus urophylla* triploids and diploids. Pathway classification of DEMs in JL (**A**) S1 (**B**) and S2 (**C**). Pathway enrichment of DEMs in JL (**D**), S1 (**E**) and S2 (**F**) between *Eucalyptus urophylla* triploids and diploids. The *Y*-axis indicates the KEGG pathway; the *X*-axis indicates the rich factor. The dot size indicates the number of DEMs of the pathway, and the dot color indicates the *p*-value

The analysis of differentially expressed metabolites (DEMs) showed that the highest number of DEMs was in JL and the lowest number of DEMs was in S1 of the triploids compared with the diploids (Fig. 4B). DEMs mainly included 11 categories, among which phenolic acids were the most abundant (Fig. 4C). According to the KEGG database annotation of DEMs, pathway classification showed that DEMs mainly participated in cofactor synthesis, amino acid synthesis, flavonoid biosynthesis and phenylpropanoid biosynthesis (Fig. 5A–C). Further pathway enrichment analysis showed that DEMs were mainly enriched in “biosynthesis of valine, leucine and isoleucine” (kegg00290), “glucosinolate biosynthesis” (kegg00966) and “flavone and flavonol biosynthesis” (kegg00944) in JL (Fig. 5D). DEMs were mainly enriched in “stilbenoid, diarylheptanoid and gingerol biosynthesis” (kegg00945), “metabolite biosynthesis of secondary metabolites” (kegg01110) and “phenylpropanoid biosynthesis” (kegg00940) in S1 (Fig. 5E). DEMs were mainly enriched in “glucosinolate biosynthesis” (kegg00966) and “biosynthesis of tropane, piperidine and pyridine alkaloid” (kegg00960) in S2 (Fig. 5F). In addition, “phenylpropanoid biosynthesis” (kegg00940) was also significantly enriched.

### Differentially expressed analysis of lignin-related metabolites between *Eucalyptus urophylla* triploids and diploids

**Fig. 6:**
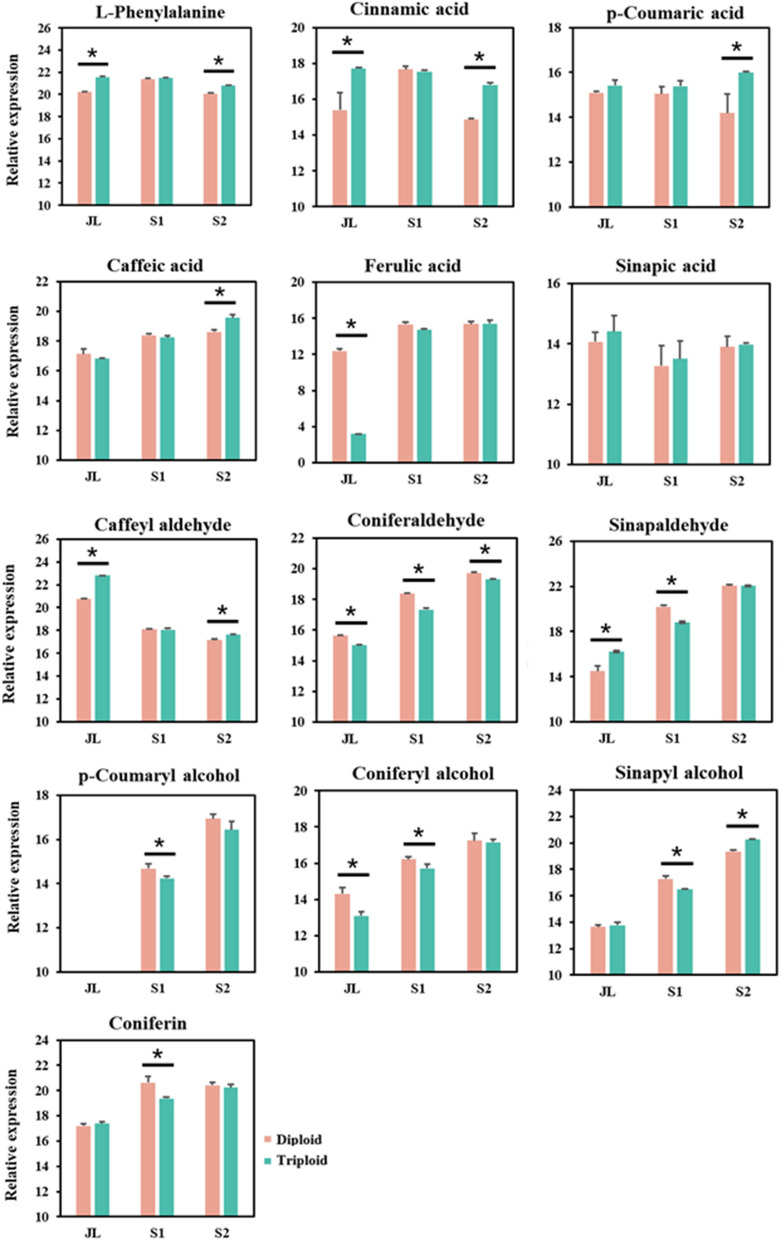
Relative expression analysis of lignin-related metabolites in *Eucalyptus urophylla* triploids and diploids. An asterisk indicates that there is a significant difference between triploids and diploids. Error bars indicate the standard deviation among three biological replicates

In this study, a total of 13 lignin-related intermediate metabolites were detected, including l-phenylalanine, cinnamic acid, *p*-coumaric acid, caffeic acid, ferulic acid, sinapic acid, caffeyl aldehyde, coniferaldehyde, sinapaldehyde, *p*-coumaryl alcohol, coniferyl alcohol, sinapyl alcohol, and coniferin (Fig. 6). l-Phenylalanine, cinnamic acid and *p*-coumaric acid are located upstream of the entire phenylpropanoid pathway. Compared with diploids, the three upstream metabolites were mainly significantly upregulated in JL and S2 of triploids. Caffeic acid was also differentially upregulated in S2 of triploids, and ferulic acid was differentially downregulated in JL of triploids. Caffeyl aldehyde was differentially upregulated in JL and S2 of triploids. Among the metabolites related to G-lignin, coniferaldehyde was significantly downregulated in the three tissues, and coniferyl alcohol was significantly downregulated in JL and S1 of triploids compared with diploids. In addition, coniferin was downregulated in S1 of triploids. Among the metabolites related to S-lignin, sinapaldehyde was significantly upregulated in JL and downregulated in S2 of triploids, and sinapyl alcohol was downregulated in S1 and upregulated in S2 of triploids compared with diploids. *p*-coumaryl alcohol, which is related to H-lignin, was significantly downregulated in S1 of triploids compared with diploids.

In general, compared with diploids, most upstream metabolites were significantly upregulated, while most downstream metabolites, especially G-lignin-related metabolites, were significantly downregulated in the triploids.

### Correlation analysis among lignin-related genes, metabolites and lignin content of *Eucalyptus urophylla* triploid and diploid stems

To identify primarily contributing metabolites during the process of lignin accumulation of triploid stems, the correlation between lignin-related metabolites and lignin content was analyzed. The results showed that phenylalanine, caffeyl aldehyde and cinnamic acid have a significant negative correlation with lignin content. Coniferaldehyde, *p*-coumaryl alcohol, sinapaldehyde and coniferyl alcohol have significant positive correlations with lignin content, indicating that they may play a more important role in influencing the final lignin accumulation of triploids (Table 1).

**Table 1:** Correlation analysis between lignin-related metabolites and lignin content

Phenotype	Metabilites	rho	*p*-value	Relation
Lignin content	Coniferaldehyde	0.921085296	0.00002	Positive
Lignin content	*p*-Coumaryl alcohol	0.840239327	0.00062	Positive
Lignin content	Sinapaldehyde	0.800569918	0.00176	Positive
Lignin content	Coniferyl alcohol	0.735416743	0.00642	Positive
Lignin content	Sinapyl alcohol	0.472616468	0.12076	Positive
Lignin content	Coniferin	0.393461906	0.20573	Positive
Lignin content	Ferulic acid	0.323517122	0.30499	Positive
Lignin content	Sinapic acid	0.238230849	0.45589	Positive
Lignin content	Caffeic acid	0.197154689	0.53910	Positive
Lignin content	*p*-Coumaric acid	0.108248022	0.73773	Positive
Lignin content	Cinnamic acid	− 0.860512245	0.00033	Negtive
Lignin content	Caffeyl aldehyde	− 0.876006818	0.00019	Negtive
Lignin content	l-Phenylalanine	− 0.911118709	0.00004	Negtive

**Fig. 7:**
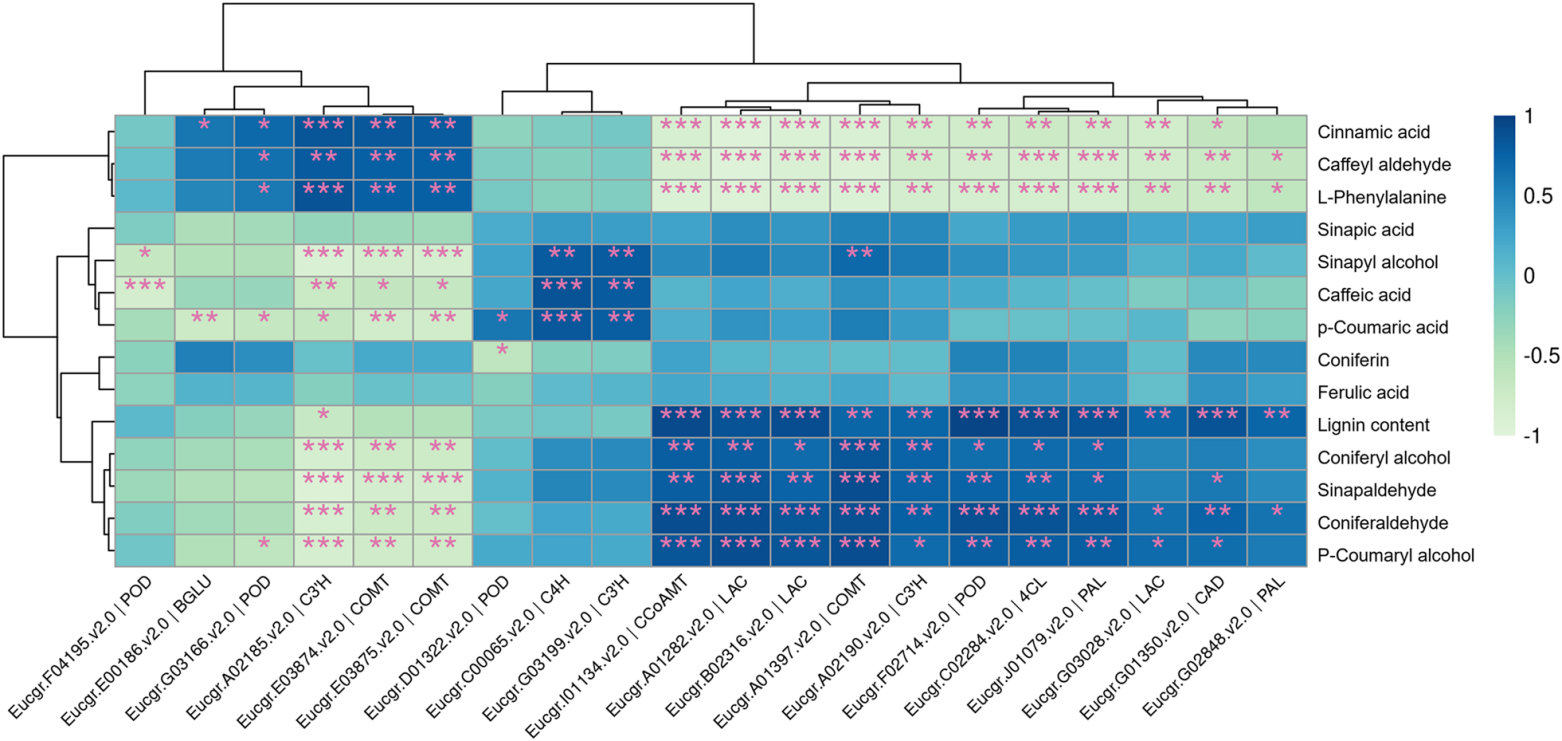
Correlation heatmap analysis of lignin-related genes, metabolites and lignin content. The color in the heatmap represents the correlation (*P < 0.05; **P < 0.01; ***P < 0.001).

We further calculated the correlations among lignin-related genes, metabolites and lignin content. The results showed that most of the genes had a significant correlation with lignin content and also had a significant positive correlation with coniferaldehyde, *p*-coumaryl alcohol, sinapaldehyde and coniferyl alcohol. A total of 12 DEGs related to lignin were significantly correlated with lignin content, among which 11 were positively correlated (Fig. 7; Additional file 6: Table S3). Therefore, these genes may play a more important role in affecting the metabolites related to lignin, and then affect the final variation of lignin content in triploids.

### Identification of key modules and genes related to lignin biosynthesis by weighted gene co-expression network analysis

**Fig. 8:**
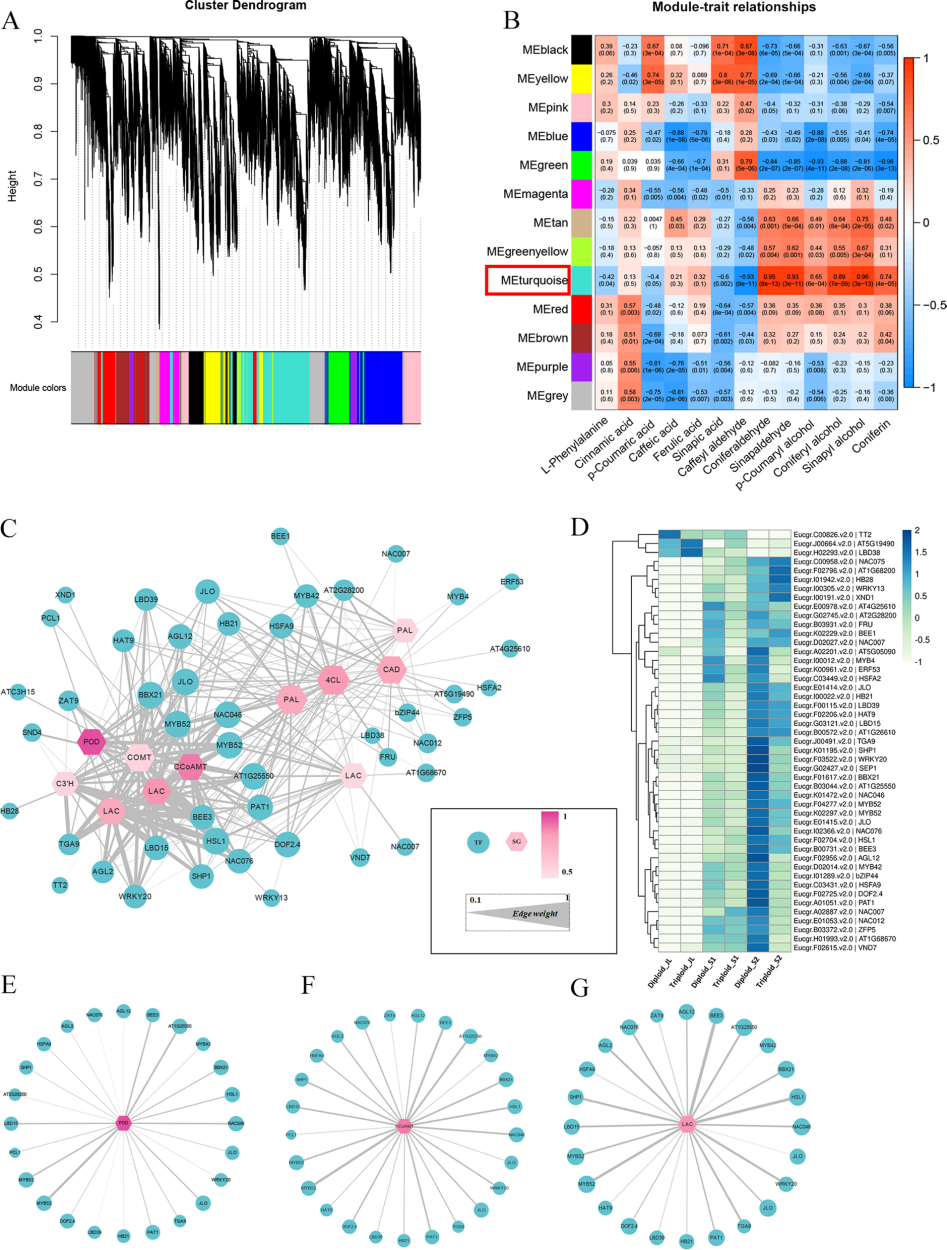
Identification of key modules and genes related to lignin biosynthesis by WGCNA. **A** Cluster dendrogram. **B** Module-trait relationships with lignin-related metabolites. **C** Co-expression network of turquoise module. The hexagon represents lignin-related SGs. Circles represent TFs. The greater the connectivity of structural genes, the darker the color of SGs. The larger the edge weight, the thicker the line. **D** Expression analysis of all TFs in the network. The values in the heatmap represent the z-score of the FPKM in different samples. The sub-network between TFs and *POD* (*Eucgr.F02714.v2.0*), *CCoAOMT* (*Eucgr.I01134.v2.0*) and *LAC* (*Eucgr.B02316.v2.0*) were visualized in **E**, **F** and **G**, respectively

Weighted gene co-expression network analysis (WGCNA) is a useful method for identifying genes with similar expression patterns that may be involved in specific biological functions [26]. To further identify important regulatory factors that may affect triploid lignin synthesis, a co-expression network between genes and metabolites was constructed. Hierarchical clustering analysis of samples showed that the repeatability among samples was consistent and that there were no outliers (Additional file 3: Figure S3). According to the standard of mixed dynamic shearing, 13 gene co-expression network modules were divided, and different colors represent different modules (Fig. 8A). Module-trait relationship analysis showed that the turquoise module was strongly correlated with the metabolites coniferaldehyde, coniferyl alcohol, sinapaldehyde and *p*-coumaryl alcohol, which had correlation coefficients of 0.95 (*p* = 8E−13), 0.89 (*p* = 7E−09), 0.93 (*p* = 3E−11) and 0.65 (*p* = 6E−04), respectively (Fig. 8B). Coniferaldehyde, coniferyl alcohol, sinapaldehyde and *p*-coumaryl alcohol were strongly correlated with lignin content. Accordingly, we carried out an in-depth analysis based on this module.

The turquoise module had 3566 genes, including 23 lignin-related genes, and it was also the module with the largest number of lignin biosynthetic genes. Among them, 11 were positively correlated with the lignin content. TFs play significant roles in regulating lignin biosynthesis. There were 191 TFs in this module, and 105 TFs were differentially expressed in at least one stem tissue. The local regulatory network of lignin biosynthesis was drawn between 11 lignin-related genes and directly linked differentially expressed transcription factors (DETFs) (weight > 0.1) (Fig. 8C). The hexagonal nodes represent lignin biosynthetic genes and the circular nodes represent TFs (Fig. 8C). The degree of connectivity to each node was represented by the node size. The color of lignin biosynthetic genes represented the correlation between the lignin content and genes, with a greater correlation corresponding to a darker node. The edge width was positively correlated with the weight between related genes. The network consisted of 11 lignin biosynthetic genes and 47 DETFs (Fig. 8C, Additional file 7: Table S4). The TFs were mainly composed of 19 TF families, among which the NAC, LBD and MYB gene families had the most members (9, 5 and 5, respectively). The expression analysis showed that most TFs were differentially downregulated in triploid stem tissues (Fig. 8D).

*POD* (*Eucgr.F02714.v2.0*), *CCoAOMT* (*Eucgr.I01134.v2.0*) and *LAC* (*Eucgr.B02316.v2.0*) had the highest correlation with the lignin content, indicating that they might have a more central role. Thus, we drew separate network diagrams between the three genes and DETFs which are shown in Fig. 8E, F and G. There were 24 DETFs directly linked to *POD*, of which *MYB52*, *HHO3* and *NAC046* were highly correlated with *POD.* There were 25 TFs directly linked to *CCoAOMT,* and the top 3 TFs with the highest correlations were *MYB52*, *BEE3* and *HHO3*. There were 24 TFs directly linked to *LAC*, among which *BEE3*, *HSL1* and *MYB52* had the highest correlation. To verify the gene expression pattern, we randomly selected several TFs in the network for the qRT-PCR analysis. The results showed that the relative expression of most genes was consistent with the transcriptome data (Additional file 2: Figure S2).

## Discussion

After polyploidization in plants, a series of phenotypic variations are usually produced under the regulation of different genetic mechanisms and can contribute to the success of polyploids in nature or their selection for use [5, 27]. In recent years, an increasing number of studies have been committed to exploring the mechanisms underlying polyploid trait variation and providing a better understanding for the utilization of polyploid resources [28, 29]. Previous studies have found that cell wall components can usually be altered in polyploids compared with diploids, among which polyploids typically have a lower lignin content than diploids, such as in herbaceous plants, including rice, sugarcane, and *Arabidopsis*, as well as shrub willow and forest trees, including poplar [6–11]. Low lignin content could be beneficial to the more efficient utilization of polyploid biomass resources. However, the mechanism underlying lignin content variation of polyploids is still unclear.

In the present study, we measured the lignin content of *Eucalyptus urophylla* triploids for the first time. The results showed that the lignin content of triploid stems was significantly lower than that of diploid stems, which is consistent with many previous studies on other species [6–11]. As the most widely planted hardwood species in the world, eucalypts are the most important sources for paper and pulp production [14]. Considering that lignin is the major limiting factor for industrial production, *Eucalyptus urophylla* triploids with a relatively low lignin content may have more application potential. Therefore, we conducted an in-depth study based on transcriptomics and metabolomics to explore the mechanism of lignin content variation in *Eucalyptus urophylla* triploids.

Metabolites, as downstream products of gene and protein expression, are more directly and closely linked to the phenotype. Plant polyploidization can often lead to different accumulation patterns of primary or secondary metabolites [30]. In this study, the majority of lignin-related metabolites detected were significantly different between triploids and diploids, suggesting that lignin accumulation had been altered at the metabolic level. The results showed that the accumulation pattern of lignin-related metabolites varied in different tissues or in different developmental stages of the same tissue. Among which, metabolites related to G-lignin, coniferaldehyde and coniferyl alcohol, might be the key metabolites influencing the difference of lignin accumulation between triploids and diploids. They had relatively consistent downregulated tendencies among the three tissues, and had significantly positive correlations with the lignin content.

DEGs usually arise with the formation of polyploids. Although the reasons for novel variations in polyploids are not fully understood, they could involve changes in gene expression [27]. Many studies have shown that changes in lignin biosynthetic genes can cause corresponding variations in lignin content or composition [31–34]. For example, antisense down-regulation of *CCoAOMT* in flax could cause the lignin content decreased and modify the lignin monomer ratio [32]. Simultaneous disruption of *LAC4*, *LAC11* and *LAC17* led to less lignification in vascular tissues in *Arabidopsis thaliana* [33]. Mutants of *AtPrx2*, *AtPrx25* and *AtPrx71* also had an influence on lignin content and plant growth in *Arabidopsis* stem [34]. Most DEGs related to lignin biosynthesis were significantly downregulated in triploid stems compared with diploids, which might cause the corresponding change of lignin content. Further analysis of these genes revealed that 11 of them were highly correlated with lignin content and key metabolites, indicating that they may have a more critical role.

To further explore the other regulatory factors that may affect lignin biosynthesis, we constructed a co-expression network between lignin biosynthetic genes and TFs within the key co-expression module based on genes and metabolites. Studies have shown that the SCW synthesis was under the regulation of a multilayered network centered on NAC and MYB TFs [20]. *NACs* (*VND1-7* and *NST1-3*) are primary switches that can regulate downstream TFs [21]. In addition, *MYBs* are the major second or third layer TFs regulating downstream SCW synthesis genes [35]. In addition, some other TF family members have also been proved to regulate lignin biosynthesis, such as LBD, WRKY and bHLH, etc. [36]. TFs as important regulatory factors participate in lignin biosynthesis, the change of their expression levels between triploids and diploids may have a certain effect on the variation of lignin content. In this network, DETFs were mainly composed of NAC and MYB TF families, some members of which have been verified to be involved in the regulation of lignin biosynthesis [20, 21, 35]. For example, two transcripts of *MYB52* were identified (*Eucgr.K02297.v2.0*; *Eucgr.F04277.v2.0*), of which *Eucgr.k02297.v2.*0 was highly expressed in stem tissues and differentially downregulated in S2 of triploids compared with diploids. *NAC076/VND2* (*Eucgr.I02366.v2.0*) was similarly highly expressed in the stems and was differentially downregulated in S2 of triploids compared with diploids. In addition, *LBD15* (*Eucgr.G03121.v2.0*) was highly expressed in stems and was differentially downregulated in S1 of triploids compared with diploids.

In the sub-network between TFs and individual lignin biosynthetic gene with high correlation with lignin content, *POD* (*Eucgr.F02714.v2.0*), *LAC* (*Eucgr.B02316.v2.0*) and *CCoAOMT* (*Eucgr.I01134.v2.0*), *MYB52 (Eucgr.K02297.v2.0), HHO3 (Eucgr.B03044.v2.0), NAC046 (Eucgr.K01472.v2.0), BEE3 (Eucgr.B00731.v2.0)* and *HSL1 (Eucgr.F02704.v2.0)* had the relatively largest edge weight. Among them, *HSL1* belongs to the B3 TF family and mainly has a repressive effect during seed maturation [37]. In the present study, *HSL1* was specifically highly expressed in stems and was differentially upregulated in S1 of triploids compared to diploids. *BEE3* belongs to the bHLH TF family, which is an early response gene for brassinosteroid [38, 39]. Its mutants could cause different expressions of genes related to the cell wall and programmed cell death [40]. *HHO3* belong to a subfamily of the G2-like TF family, and studies have shown that *HHO3* play an important role in N and P absorption and utilization, plant growth and development, and abiotic stress [41]. *NAC046* is a member of the NAC domain-containing family of TFs, which regulate chlorophyll degradation and plant senescence and are also involved in suberine synthesis, programmed cell death, etc. [42, 43]. Compared with diploids, these TFs with high edge weights, such as *BEE3*, *NAC046*, *HSL1*, *HHO3*, had significantly differences in triploid stem tissues, which could be new potential regulatory factors for lignin biosynthesis and were valuable for further analysis to explore the functions, such as by means of overexpression or knockout.

In the study, through analyzing lignin-related metabolites, lignin biosynthetic genes and TFs, we found some of which could be relatively sensitive to genome-wide doubling effects, accompanying significant differences in the expression levels between triploids and diploids. These factors significantly responsive to polyploidization that may play a more crucial role in leading to the variation of lignin content in *Eucalyptus urophylla* triploids. This study provides a more comprehensive understanding of the underlying mechanism of polyploid lignin–trait variation, which contributes to the utilization of polyploid biomass resources. In addition, it is valuable for further studying these candidate regulatory factors, which will provide important targets for improving/reducing lignin content by molecular technologies to genetic improvement.

## Conclusion

After plant polyploidization, the gene expressions of polyploids usually change and cause corresponding phenotypic variations. In this study, the lignin content of *Eucalyptus urophylla* triploid stems was significantly reduced. This variation could be beneficial to use polyploid biomass resources for industrial production, such as for pulp, paper and biofuel. An integrated multi-omics analysis showed that lignin-related metabolites of triploids, such as coniferaldehyde and coniferyl alcohol, were differentially accumulated with highest correlation with lignin content. Most lignin biosynthetic genes were differentially downregulated, such as *PAL*, *4CL*, *C3*′*H*, *COMT*, *CCoAOMT*, *CAD*, *LAC*, *POD*. In addition, several transcription factors highly co-expressed with lignin biosynthetic genes including* MYB52*, *NAC076/VND2*, *LBD15*, *BEE3*, *NAC046*, *HSL1* and *HHO3*, were also identified, which may be involved in regulating triploid lignin synthesis. The significant changes of these key structural genes, regulatory factors and metabolites between *Eucalyptus urophylla* triploids and diploids may together lead to the variation of lignin accumulation.

## Materials and methods

### Plant materials

Clones of triploid (2n = 3× = 33) and its diploid parents (2n = 2× = 22) of *Eucalyptus urophylla* were used in this study. This triploid (clone number EU17) was an autotriploid which was self-crossing through 2n female gamete and the haploid male gamete provided by only diploid parent (clone number EU18). The technique for inducing 2n female gametes to produce triploid was derived from previous study [15]. Polyploid progeny was tested by flow cytometer and somatic chromosome counting. All plantlets were planted with the same environment at the Guangxi Dongmen Forest Farm (Guangxi Zhuang Autonomous Region, China). Rooted plantlets from a tissue culture of triploids and diploids of the same age were transplanted separately into the same nutrient cup at the same time. After approximately 120 days, nine seedlings were, respectively, selected from the two groups of clones and, respectively, divided into three groups as three replicates to conduct related experiments. The top 5 cm of the first stem internode was collected and is represented by S1, the upper 5 cm of the second stem internode was collected and is represented by S2, and the juvenile leaves were collected and are represented by JL.

## Lignin content determination

Stems from 4-month-old *Eucalyptus* triploids and diploids were collected and dried at 105 ℃. The dried tissues were ground to a fine powder by ball milling and used to prepare alcohol insoluble residues (AIRs) according to Foster [44]. The lignin content was determined according to Sluiter [45]. In brief, 300 mg of AIRs was weighed into test bottles, and 3 mL 72% sulfuric acid was added at 30 ℃ for 60 min, with vortexing every 5 min. Then, distilled water was added to dilute the solution to 3%, and the mixture was allowed to react at 120 ℃ for 20 min. After cooling to room temperature, the solution was filtered by glass crucibles and dried at 105 ℃ to a constant weight. The lignin content was calculated according to the difference in weight before and after crucible drying. Three replicates of each ploidy were performed in this study.

### Histochemical staining

Stems from 4-month-old *Eucalyptus* triploids and diploids were collected and sectioned for histochemical assays. The cross sections were stained with 1% phloroglucinol (w/v) in 12% HCl for 5 min and immediately observed and photographed under a microscope (BX51, Olympus) equipped with a CCD camera (DP70; Olympus) [46].

### Transcriptome sequencing and analysis

Total RNA was extracted using TRIzol reagent (Invitrogen, Carlsbad, CA, USA) following the manufacturer's procedure. The RNA amount and purity of each sample were quantified using a NanoDrop ND-1000 (NanoDrop, Wilmington, DE, USA). The RNA integrity was assessed by a Bioanalyzer 2100 (Agilent, Palo Alto, CA, USA) and confirmed by electrophoresis with a denaturing agarose gel. Oligo (dT) magnetic beads (Dynabeads Oligo (dT), No. 25-61,005, Thermo Fisher, USA) were used to specifically capture mRNA containing PolyA (polyadenylate) through two rounds of purification. The captured mRNA was fragmented using the NEBNext^®^ Magnesium RNA Fragmentation Module (item number E6150S, USA) under high temperature conditions at 94 ℃ for 5–7 min. cDNA was synthesized from the fragmented RNA using Invitrogen SuperScript^™^ II Reverse Transcriptase (item No. 1896649, CA, USA). Finally, we used illumina Novaseq^™^ 6000 (LC Bio Technology CO.,Ltd. Hangzhou, China) to double-end sequencing in PE150 mode, as per standard procedure. The reads containing adaptor contamination were removed to obtain clean reads. Clean reads were mapped to the genome (*Eucalyptus grandis* v2.0) using HISAT2 software [12, 47]. The mapped reads of each sample were assembled and all transcripts were merged to reconstruct a comprehensive transcriptome [48]. The expression levels of all transcripts were estimated by calculating the fragments per kilobase of transcript per million mapped reads (FPKM). The DEGs were selected based on fold change ≥ 1.5 or ≤ 0.67 and false discovery rate < 0.05 by edgeR [49]. The pathway enrichment analysis was performed by mapping DEGs to the Kyoto Encyclopedia of Genes and Genomes (KEGG) database (http://www.kegg.jp/) [50].

### Quantitative real-time PCR

The relative expression pattern of genes in transcriptome data were analyzed by qRT-PCR. Total RNA was extracted using TRIzol reagent (Invitrogen, Carlsbad, CA, USA) following the manufacturer's procedure. cDNA was synthesized using a FastQuant RT Kit (with gDNase) (Tiangen Biotech co., LTD, Beijing, China) following the manufacturer’s instructions. The primers for the genes were designed using Primer 5.0 software and are listed in Additional file 4: Table S1. The reference gene used was *EuGADPH*. qRT-PCR was performed with a SuperReal PreMix Plus (SYBR Green) kit (TransGen Biotech, Beijing, China) according to the manufacturer’s recommendation using an Applied Biosystems 7500 Fast Instrument (AB Ltd., Lincoln, NE, USA). The relative expression was calculated using the 2^−ΔΔCT^ method. Three biological and three technical replicates were performed in this assay.

### Metabolomic analysis

Metabolite profiling was carried out using a widely targeted metabolome method by Wuhan Metware Biotechnology Co., Ltd. (Wuhan, China). The samples used for metabolite profiling were the same as those used for transcriptome sequencing. Biological samples are freeze-dried by vacuum freeze-dryer (Scientz-100F) and crushed using a mixer mill (MM 400, Retsch). Sample powders were extracted with 70% methanol solution at 4 ℃ overnight. Following centrifugation, the extracts were filtrated and analyzed using an UPLC–ESI–MS/MS system (HPLC, Shimpack UFLC SHIMADZU CBM30A system; MS, Applied Biosystems 6500 Q TRAP). The analytical conditions were according to standard procedures of MetWare (Wuhan, China). The metabolite quantification was based on metware database by MetWare (Wuhan, China). The metabolite quantification was using multiple reaction monitoring (MRN) model. After obtaining the mass spectrum data of different substances, the peak area of the mass spectrum peaks of all substances was calculated, and the mass spectrum peaks of the same metabolite in different samples were integrated and corrected.

The significantly different metabolite between groups was analyzed based on orthogonal partial least squares discriminant analysis (OPLS-DA), A threshold of variable importance in projection (VIP) value ≥ 1 and |fold change| ≥ 1.5 were deemed to differentially expressed metabolites (DEMs). DEMs were annotated using the KEGG compound database (http://www.kegg.jp/kegg/compound/). Annotated metabolites were then mapped to the KEGG pathway database (http://www.kegg.jp/kegg/pathway.html). Pathways with significantly regulated metabolites mapped to were then fed into MSEA (metabolite sets enrichment analysis), their significance was determined by hypergeometric test’s *p* values.

### Correlation analysis

The correlation among genes, metabolites and lignin content was calculated in this study. The FPKM of genes, peak areas of metabolites and lignin content were used for Pearson correlation analysis using the R package (‘Stats’). For *p* values < 0.05, the correlation was deemed significant.

### Weighted gene co-expression network analysis

After filtering genes with low expression (the sum of all sample expression < 10), a total of 17,784 genes were assessed via weighted gene co-expression network analysis (WGCNA) using the R package WGCNA. The weighted co-expression modules were constructed with the following parameters: weighted network = unsigned, power = 25, minimum module size = 50, minimum height for merging modules = 0.25, and deepSplit = 2. Pearson’s correlation coefficient was used to determine the relationship between modules and lignin-related metabolites (l-phenylalanine, cinnamic acid, *p*-coumaric acid, caffeic acid, ferulic acid, sinapic acid, caffeyl aldehyde, *p*-coumaryl alcohol, coniferaldehyde, coniferyl alcohol, sinapaldehyde, sinapyl alcohol, and coniferin) among 18 samples. The co-expression network was constructed according to the edge weights between genes and visualized by Cytoscape 3.9.0 software.

## Supplementary Information

**Additional file 1: Figure S1.** Validation of the expression pattern of lignin biosynthetic genes by qRT-PCR.

**Additional file 2: Figure S2. **Validation of the expression pattern of transcription factors by qRT-PCR.

**Additional file 3: Figure S3.** Cluster analysis of samples in the co-expression network.

**Additional file 4: Table S1.** Primer sequences of genes in qRT-PCR.

**Additional file 5: Table S2.** Statistical analysis of each sample in the transcriptomic data.

**Additional file 6: Table S3.** Correlation of lignin-related genes, metabolites and lignin content.

**Additional file 7: Table S4. **Degree of transcription factors in the co-expression network.

## Data Availability

All data generated or analyzed during this study are included in this published article and its supplementary information files. The clones used in this study are planted and stored in the State Key Laboratory of Tree Genetics and Breeding, Beijing Forestry University (Beijing, China).

## Abbreviations

SCWs: Secondary cell walls
DEGs: Differentially expressed genes
TFs: Transcription factors
AIRs: Alcohol insoluble residues
FPKMs: Fragments per kilobase of transcript per million mapped reads
KEGG: Kyoto Encyclopedia of Genes and Genomes
OPLS-DA: Orthogonal partial least squares discriminant analysis
VIP: Variable importance in projection
WGCNA: Weighted gene co-expression network analysis
DEMs: Differentially expressed metabolites
