# Supplementary figures and images for: Integrated transcriptomic and metabolomic analysis reveals the effects of polyploidization on the lignin content and metabolic pathway in Eucalyptus
Source: Biotechnol Biofuels Bioprod. 2023 Jul 21;16:117. doi: 10.1186/s13068-023-02366-4 (PMC10360242; doi:10.1186/s13068-023-02366-4)

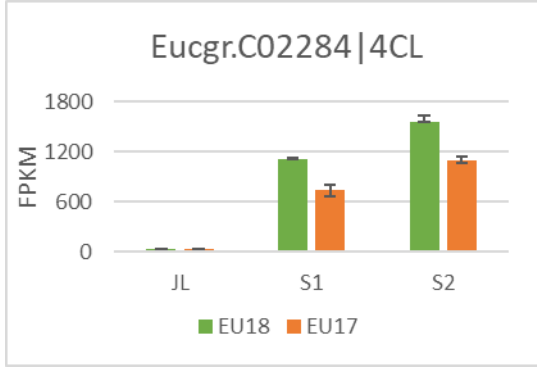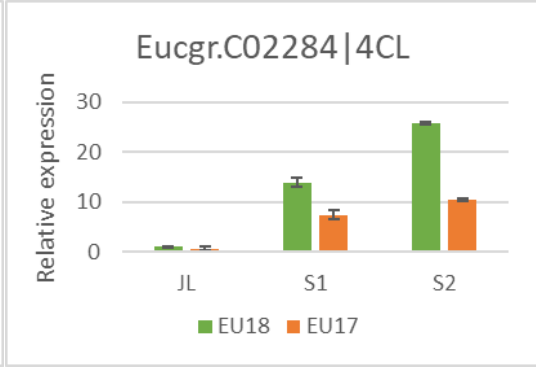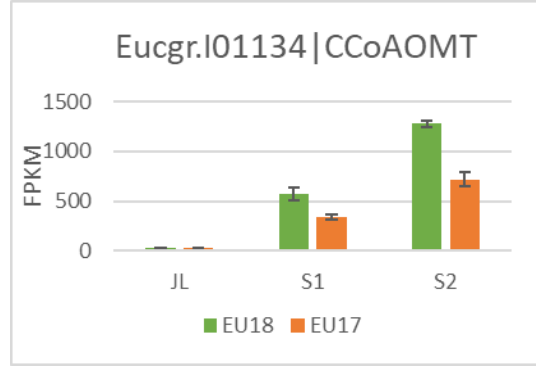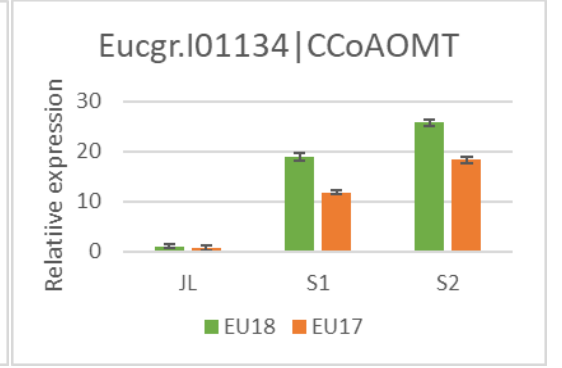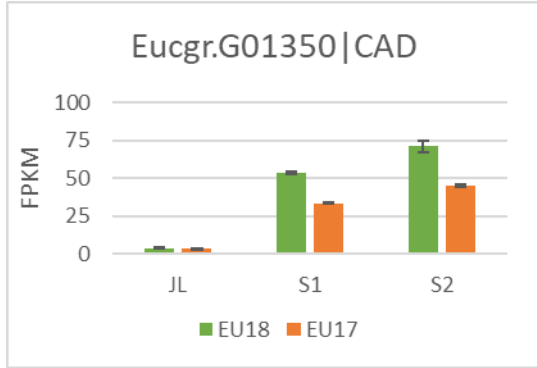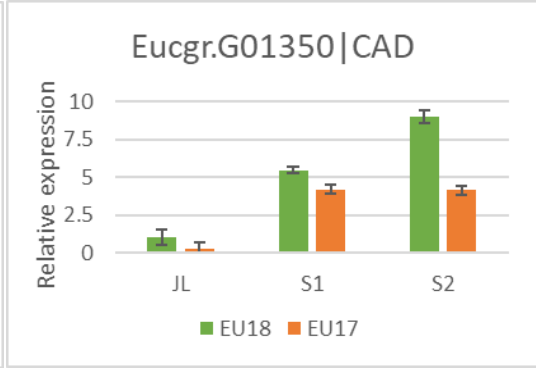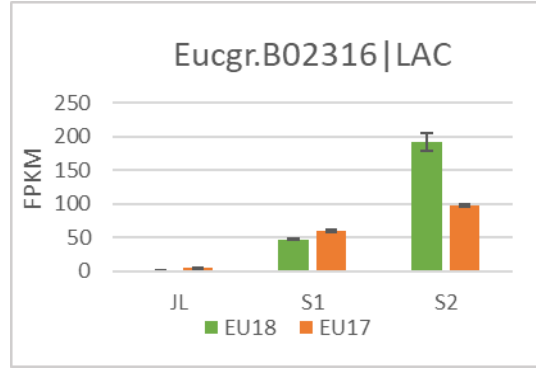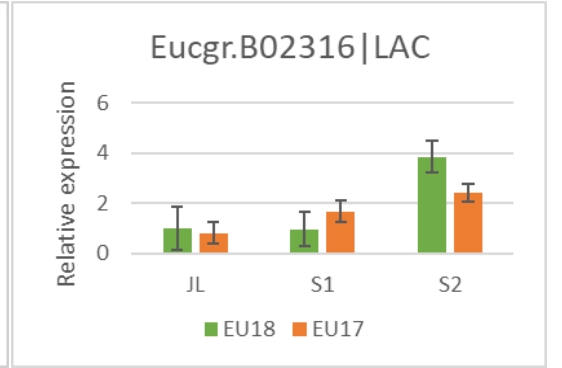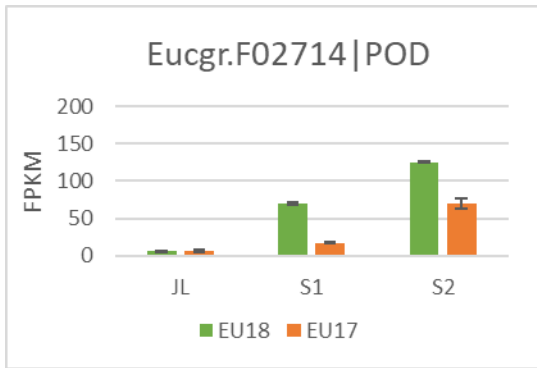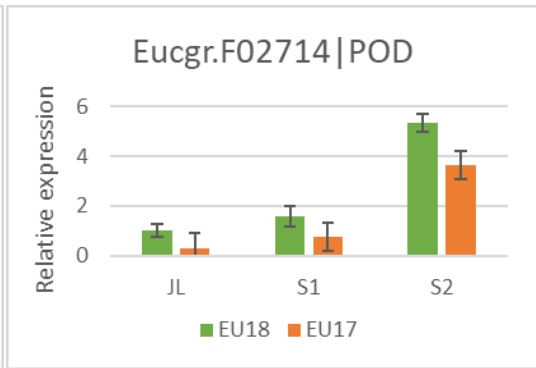

Supplement: Supplementary file 1 — Additional file 1: Figure S1. Validation of the expression pattern of lignin biosynthetic genes by qRT-PCR. [file 13068_2023_2366_MOESM1_ESM.pdf]

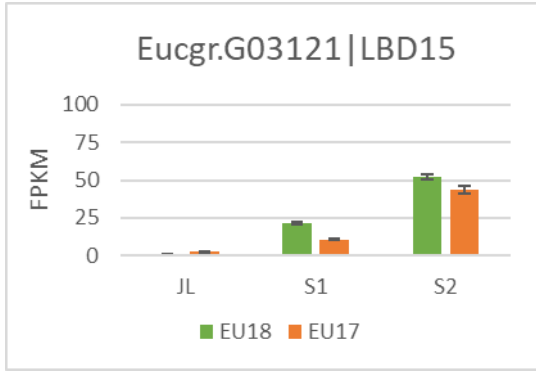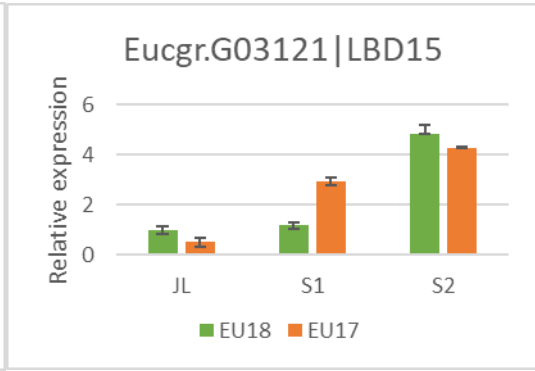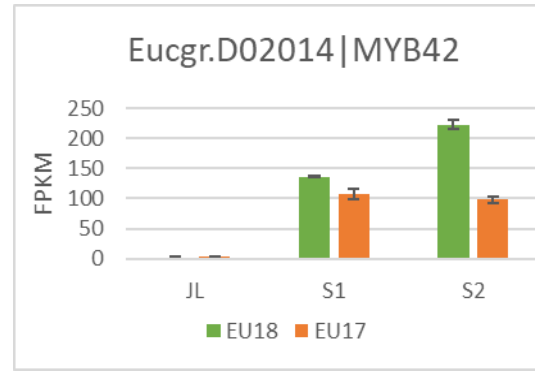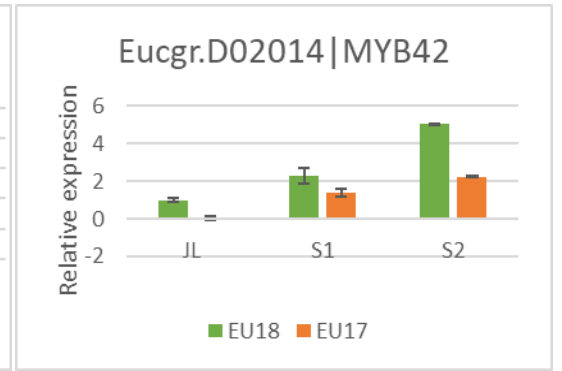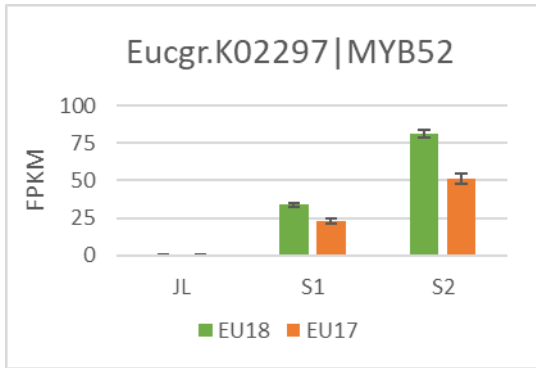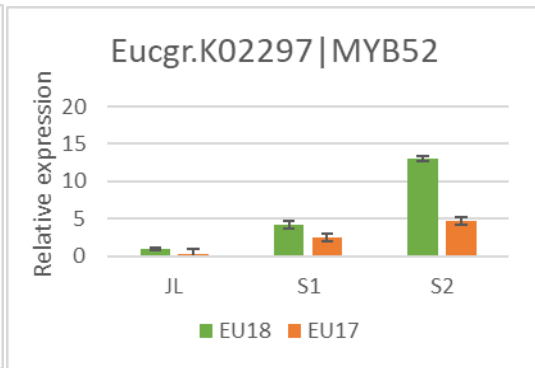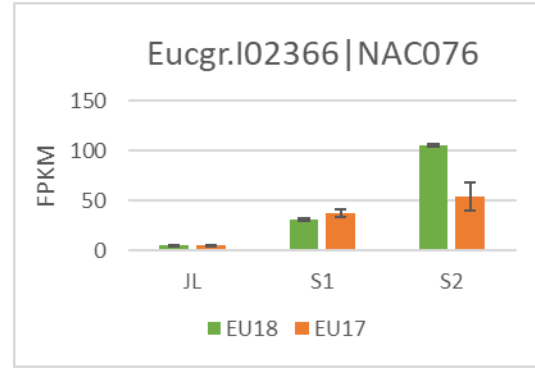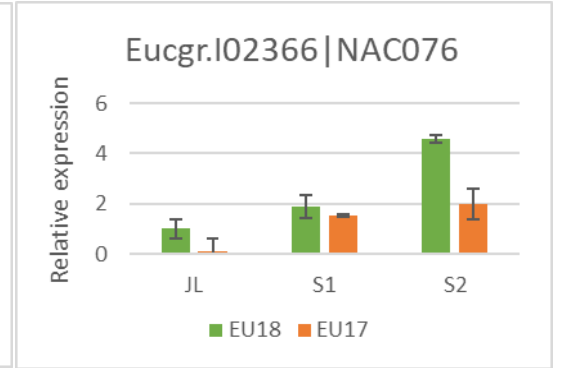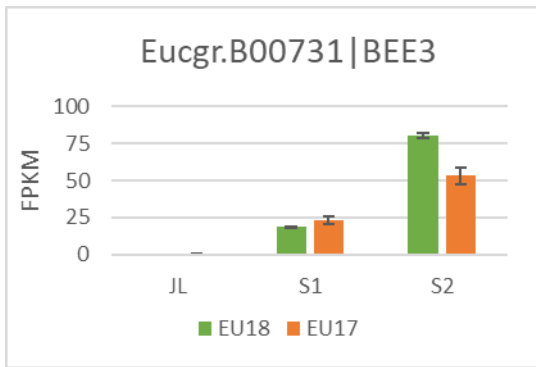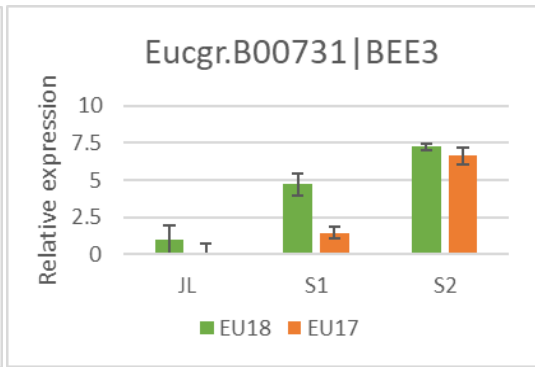

Supplement: Supplementary file 2 — Additional file 2: Figure S2. Validation of the expression pattern of transcription factors by qRT-PCR. [file 13068_2023_2366_MOESM2_ESM.pdf]

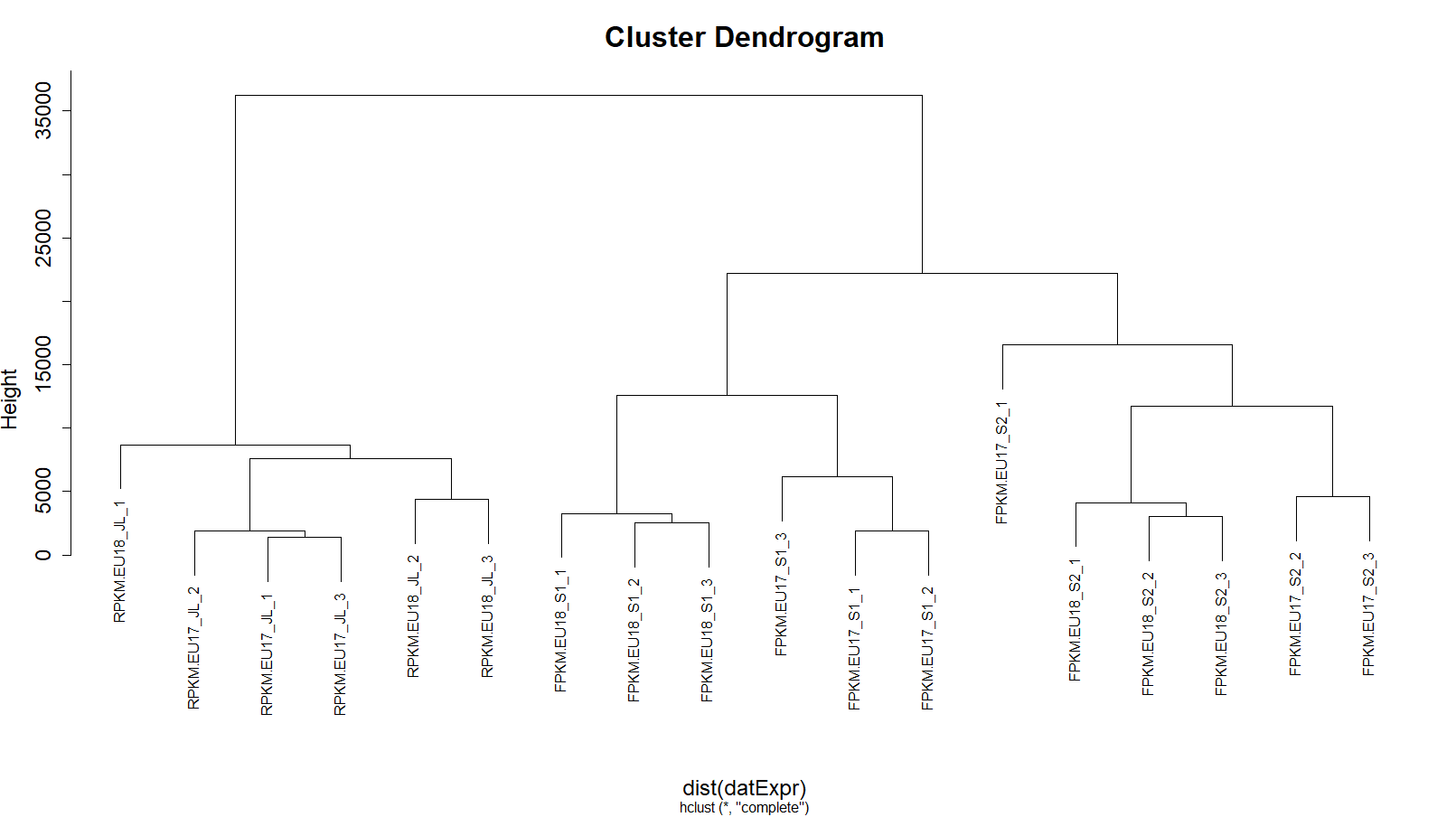

Supplement: Supplementary file 3 — Additional file 3: Figure S3. Cluster analysis of samples in the co-expression network. [file 13068_2023_2366_MOESM3_ESM.tiff]
